# Simultaneous Intra- and Extracochlear Electrocochleography During Cochlear Implantation to Enhance Response Interpretation

**DOI:** 10.1177/2331216521990594

**Published:** 2021-03-12

**Authors:** Leanne Sijgers, Flurin Pfiffner, Julian Grosse, Norbert Dillier, Kanthaiah Koka, Christof Röösli, Alexander Huber, Adrian Dalbert

**Affiliations:** 1 University of Zurich, Zurich, Switzerland; 2 Department of Otorhinolaryngology-Head and Neck Surgery, University Hospital of Zurich, Zurich, Switzerland; 3 Research and Technology, Advanced Bionics LLC, Santa Clarita, California, United States

**Keywords:** cochlear implant, electrocochleography, hearing preservation, residual hearing

## Abstract

The use of electrocochleography (ECochG) for providing real-time feedback of cochlear function during cochlear implantation is receiving increased attention for preventing cochlear trauma and preserving residual hearing. Although various studies investigated the relationship between intra-operative ECochG measurements and surgical outcomes in recent years, the limited interpretability of ECochG response changes leads to conflicting study results and prevents the adoption of this method for clinical use. Specifically, the movement of the recording electrode with respect to the different signal generators in intracochlear recordings makes the interpretation of signal changes with respect to cochlear trauma difficult. Here, we demonstrate that comparison of ECochG signals recorded simultaneously from intracochlear locations and from a fixed extracochlear location can potentially allow a differentiation between traumatic and atraumatic signal changes in intracochlear recordings. We measured ECochG responses to 500 Hz tone bursts with alternating starting phases during cochlear implant insertions in six human cochlear implant recipients. Our results show that an amplitude decrease with associated near 180° phase shift and harmonic distortions in the intracochlear difference curve during the first half of insertion was not accompanied by a decrease in the extracochlear difference curve’s amplitude (*n *= 1), while late amplitude decreases in intracochlear difference curves (near full insertion, *n* = 2) did correspond to extracochlear amplitude decreases. These findings suggest a role for phase shifts, harmonic distortions, and recording location in interpreting intracochlear ECochG responses.

## Introduction

Minimizing cochlear trauma during insertion of a cochlear implant (CI) has become a goal in recent years, as both the initial trauma and the intracochlear tissue response to trauma lead to reduced CI functionality as well as a loss of residual hearing when present. Electrocochleography (ECochG) is a promising method to objectively measure changes in cochlear function during cochlear implantation. It relies on the underlying assumption that damage or contact to cochlear structures causes an immediate reduction in electrophysiological signals generated by the cochlea in response to sound, and that this signal reduction can be detected in measurements acquired during surgery. In recent years, our group and others have been studying intraoperative ECochG measurements and their relationship to hearing outcomes and surgical trauma. The eventual aim is to use ECochG for intraoperative real-time feedback about cochlear function and thereby prevent cochlear trauma.

The ECochG response can be subdivided into four components: the cochlear microphonic (CM), the auditory nerve neurophonic (ANN), the compound action potential, and the summating potential. The CM seems to be the most promising ECochG component for monitoring cochlear trauma intraoperatively, as it appears to be the most sensitive to cochlear trauma (Choudhury et al., 2011) and can be recorded in 95% of CI recipients with pre-operative residual hearing (Dalbert et al., 2018). It is often assumed that the CM equals the difference between two ECochG recordings with inverted starting phases as the subtraction would cancel out the ANN components in both signals. The ANN would then be isolated by adding two alternating-polarity ECochG recordings. However, these assumptions are not valid at the low frequencies and high intensities normally used for measuring ECochG signals in CI recipients due to nonlinear effects (Forgues et al., 2014). Therefore, it has become common practice to calculate the sum and difference curves from alternating-polarity ECochG recordings without further specifying their origins; the difference signal represents mostly hair cell (CM) components and the sum signal largely consists of the ANN, compound action potential, and summating potential.

ECochG signals can be recorded either from a fixed position near the cochlea (extracochlear ECochG) or from the CI electrode array (intracochlear ECochG). In recent years, various studies investigated intraoperative ECochG measurements in human CI recipients, acquired from intracochlear locations (Acharya et al., 2016; Bester et al., 2017; Calloway et al., 2014; Campbell et al., 2015, 2016; Giardina et al., 2019; Harris, Riggs, Giardina, et al., 2017; Harris, Riggs, Koka, et al., 2017; Koka et al., 2018; Mandalà et al., 2012; O’Connell et al., 2017; Ramos-Macias et al., 2019; Riggs et al., 2019; Scott et al., 2016), extracochlear locations (Adunka et al., 2016; Dalbert et al., 2016; Dalbert, Sim, et al., 2015; Giardina et al., 2018; Haumann et al., 2019; Radeloff et al., 2012; Scott et al., 2016), or from both (Dalbert et al., 2018; Dalbert, Pfiffner, et al., 2015; Dalbert, Rohner, et al., 2020). Although several studies report relationships between ECochG measurements and surgical outcomes, measured either in terms of hearing outcomes or electrode array positioning, these relationships are not consistent among studies. Our research group published three articles demonstrating a correlation between amplitude decreases of extracochlear ECochG recordings and postoperative hearing loss (Dalbert et al., 2016, 2018; Dalbert, Sim, et al., 2015), while Adunka et al. (2016) and Haumann et al. (2019) found no such correlation. With respect to intracochlear ECochG, a response decrease was shown to relate to postoperative acoustic hearing loss by Campbell et al. (2016), Acharya et al. (2016), Dalbert et al. (2018) and Giardina et al. (2019), but not by O’Connell et al. (2017). It must be noted that response amplitude decreases can also result from reversible changes in basilar membrane mechanics, for example due to contact between the electrode array and the basilar membrane (DeMason et al., 2012).

Recording ECochG signals from locations within the cochlea has benefits compared to measuring ECochG responses extracochlearly. First, intracochlear responses have larger amplitudes, resulting in better signal-to-noise ratios and therefore shorter acquisition times (Calloway et al., 2014; Dalbert, Pfiffner, et al., 2015). Second, in intracochlear measurements, the recording electrode is closer to the apical region of the cochlea relevant for low-frequency residual hearing as well as to the location of possible damage, which may make intracochlear measurements more sensitive to direct cochlear trauma (Dalbert, Rohner, et al., 2020). In extracochlear measurements, the spread of fluid mixing may take some time to reach the population of response generators in case of a relatively apical translocation, where the response generators may lie more basally. However, the intracochlear ECochG response is more complex and less well understood than the extracochlear ECochG response. When measuring from the tip of the CI electrode array, the recording electrode could move past different signal generators. This may cause amplitude drops unrelated to cochlear trauma, making interpretation of intracochlear ECochG signals with respect to cochlear trauma difficult (Giardina et al., 2018).

Two methods for acquiring intracochlear ECochG signals are currently in use. One approach uses the CI itself to digitize and transfer the measured signal, utilizing the circuits normally used for measuring electrical impedances and neural responses during routine clinical measurements. Measurements are usually obtained using software provided by the CI manufacturer. Responses acquired using this method are affected by limited gain and a short recording window, influencing both the sensitivity to cochlear damage and the recording duration. The other approach, previously used by Harris, Riggs, Koka, et al. (2017) and Giardina et al. (2019), uses a clip electrode in the surgical field connected to the CI’s extracochlear reference ring electrode, with software to connect the most apical intracochlear electrode to the reference ring electrode. This method allows for connecting the clip electrode used for signal acquisition to any recording hardware, thus allowing measurement by the same equipment used for recording extracochlear ECochG signals.

A better understanding of the generators contributing to the intracochlear ECochG response is needed for distinguishing response decreases related to cochlear damage and response decreases solely resulting from a change in intracochlear recording location during electrode array insertion. Recently, our group conducted a study comparing simultaneous intra- and extracochlear ECochG recordings in atraumatic electrode insertions with a short, custom-made electrode (Dalbert, Sijgers, et al., 2020). The aim was to characterize ECochG signal changes solely occurring due to electrode movements. This study compares simultaneous intra- and extracochlear ECochG responses obtained over the entire insertion trajectory of a CI electrode array, including both atraumatic and most likely traumatic insertions. It thereby aims to characterize patterns in intracochlear ECochG recordings and their correspondence to extracochlear ECochG patterns while also exploring possible mechanisms underlying intracochlear ECochG changes. This is the first report of simultaneous intra- and extracochlear ECochG recordings during standard CI surgery.

## Methods

Adult subjects with residual hearing undergoing CI surgery at the CI center of the University Hospital of Zurich were enrolled in this study. As the goal was to explore the use of ECochG as a general tool for insertion monitoring, subjects with any degree of residual hearing were included, regardless of whether these subjects would be candidates for electro-acoustic stimulation or not. The study was performed with the approval of the Ethical Committee of Zurich (KEK-ZH 2013-0317) and in concordance with the Helsinki Declaration. All subjects provided written informed consent before surgery. Subject S119 received a HiFocusV CI (Advanced Bionics LLC, Stäfa, Switzerland), and all other subjects received a SlimJ model from the same manufacturer. Pure-tone audiograms were conducted within 3 months prior to surgery and approximately 4 weeks after surgery and were performed according to ISO 8235-1. Air conduction threshold values were determined at 0.125, 0.25, 0.5, 1, and 2 kHz. To calculate the pure-tone average from these frequencies, the maximum output of the audiometer plus 5 dB was used as a threshold value if no response was present at the maximum output of the audiometer. Three hearing preservation categories were defined based on the pre- and postoperative pure tone thresholds (Balkany et al., 2006): (a) complete hearing preservation (mean low-frequency hearing loss of ≤ 10 dB), (b) partial hearing preservation (mean low-frequency hearing loss of >10 dB with some remaining low-frequency hearing), and (c) no hearing preservation (complete loss of residual hearing). In all subjects except S119, a postoperative computed tomography (CT) scan was made within 1 to 3 days after surgery. In S119, an intra-operative digital volume tomography (DVT) scan was performed.

### Measurement Protocol Within the Surgical Workflow

**Figure 1.**
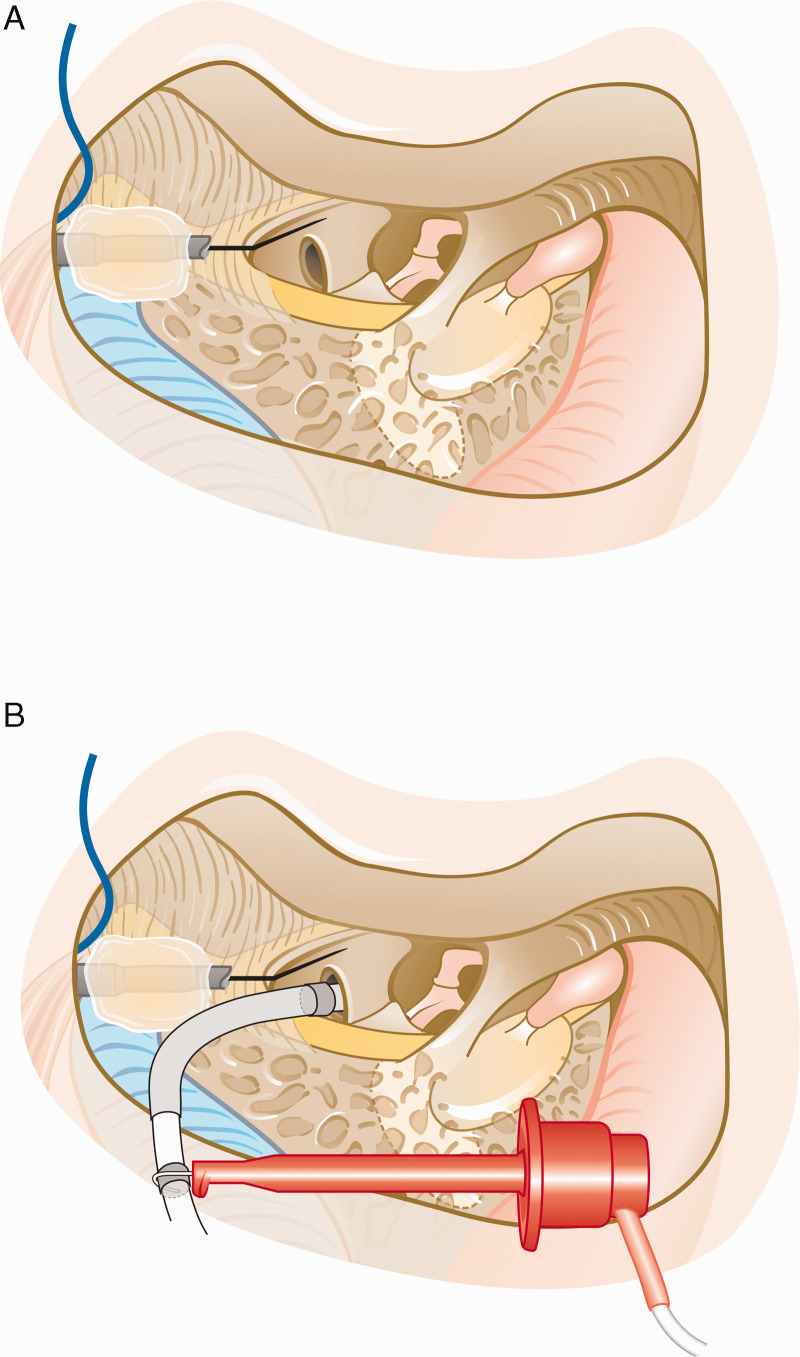
Sketch of the recording setup. A: Sketch of the surgical view with the extracochlear needle electrode placed on the promontory and attached to the mastoidectomy cavity using bone wax. B: Sketch of the same setup after inserting the CI’s electrode array into the cochlea and attaching the clip electrode to the extracochlear reference ring electrode of the CI for recording intracochlear signals.

Before surgery, an insert earphone (Biologic Systems, Mundelein, IL, United States) and a probe microphone (ER-7C, Etymotic, Inc., Elk Grove Village, IL, United States) were placed in the ear canal. Needle electrodes (20 × 0.3 mm, Neurosign, Magstim Co., Wales, United Kingdom) were placed in the contralateral preauricular region (negative) and on the forehead (ground). Next, an anterior mastoidectomy and posterior tympanotomy were performed, and the round window was completely visualized. Then, a 500 Hz acoustic signal at 100 dB SPL was routed to the insert earphone and the resulting sound pressure in the ear canal was measured using the probe microphone and visualized using an oscilloscope to verify the sound pressure close to the tympanic membrane. After verification of the sound pressure, a needle electrode (20 × 0.3 mm, Neurosign, Magstim Co.) was placed near the round window for extracochlear ECochG recordings (Figure 1A).

Once impedances were <10 kΩ on all electrodes, baseline extracochlear ECochG recordings were performed. The acoustic signal level was gradually increased until a clear ECochG response could be measured (except for subject S127, whose first extracochlear ECochG recording was performed after opening the round window). Then, the round window membrane was incised and ECochG recordings were repeated (subjects S14, S125, S126, and S127). Next, the clip electrode was attached to the reference ring electrode of the CI which was shorted to the most apical electrode of the CI electrode array using custom software from Advanced Bionics Corporation. The first two electrodes of the CI were then inserted into the cochlea (see Figure 1B) and the first simultaneous ECochG recording was made from the extracochlear electrode and the clip electrode functioning as an intracochlear recording electrode. For further simultaneous recordings, the CI was inserted in a stepwise manner to allow for recording ECochG signals while holding the electrode array in place. The CI insertion depth during each recording was marked and video recorded, such that the location of the intracochlear recording electrode could afterwards be estimated using specifications provided by the manufacturer. After full insertion, the final ECochG measurements were made. After closing the round window, the insert earphone was disconnected from the loudspeaker and a simultaneous ECochG recording was repeated for assessment of the noise floor (except for subject S126, in whom the intracochlear electrode was removed before performing the noise measurement). Finally, the intra- and extracochlear recording electrodes were removed and the surgical wound was closed.

### ECochG Measurement Specifics

The Navigator Pro stimulation/recording device (Biologic Systems, Mundelein, IL, United States) was used for acoustic stimulation and simultaneous recordings of intra- and extracochlear ECochG signals. For acoustic stimulation, a 500 Hz tone was used with a plateau phase of 10 cycles and an additional 2-cycle rise and fall time shaped by a Blackman window. The total stimulus duration was 28 ms. The sound intensity was 120 dB SPL for all recordings in all patients except for S122, for whom a sound intensity of 110 dB SPL was used for all recordings. The ECochG responses were recorded at a sampling rate of 8000 Hz with a recording window of 32 ms, starting 4 ms before stimulus presentation. At each recording location, responses to 400 acoustic stimuli with alternating starting phases were acquired and band-pass filtered with the high-pass filter cutoff frequency set at 10 Hz and the low-pass filter cutoff frequency set at 3000 Hz. Separate averages were obtained for condensation and rarefaction stimuli.

### Data Analysis

To export data from the Auditory Evoked Potential software (Biologic Systems), the Auditory Evoked Potential to ASCII software (Biologic Systems) was used. The data were analyzed using MATLAB (MathWorks, Inc., Natick, MA, United States). Difference curves were obtained by subtracting the averaged rarefaction response from the averaged condensation response; condensation and rarefaction averages were added to obtain the sum curves. A Fast Fourier Transform (FFT) was performed on the difference and sum curves using a rectangular window over the range of 8 to 24 ms. The amplitude and phase of each difference curve were obtained from the FFT bin at 500 Hz; the amplitude of the sum curve was determined from the FFT amplitude at 1 kHz. The reason for looking at the phase of the difference curves was that it could reflect underlying mechanisms, such as a change in generators underlying the measured signals, which may be relevant when using ECochG as a monitoring tool intra-operatively (Giardina et al., 2019).

In a previous study (Dalbert et al., 2016), ECochG responses recorded under unchanged conditions showed a mean difference of 0.1 dB with a standard deviation of 1.2 dB. Therefore, a change of at least 3 dB (>2 standard deviations) in difference curve amplitudes or sum curve amplitudes during the track was considered relevant here. A measurement was considered valid if its FFT amplitude was at least 6 dB above the amplitude of the corresponding FFT bin in the noise-floor recording. A value of 6 dB was chosen because this equals twice the amplitude change that is considered relevant. For subject S126, as no intracochlear noise-floor recording was made, the average intracochlear noise amplitude calculated over all other subjects was taken. An additional requirement for the sum curves was that the amplitude had to be at least −25 dB relative to 1 µV to ensure that all valid recordings were also visually detectable above the noise floor.

An experienced otorhinolaryngologist (A.D.) assessed the scalar localization and insertion angle of the CI electrode array on postoperative clinical CT scans. Comparisons were made between the amplitudes of the intra- and extracochlear sum and difference curves as well as for phase changes in the intra- and extracochlear difference curves. Changes in intra- and extracochlear difference and sum curves were compared with hearing outcomes, electrode positioning, and recording locations.

## Results

**Figure 2.**
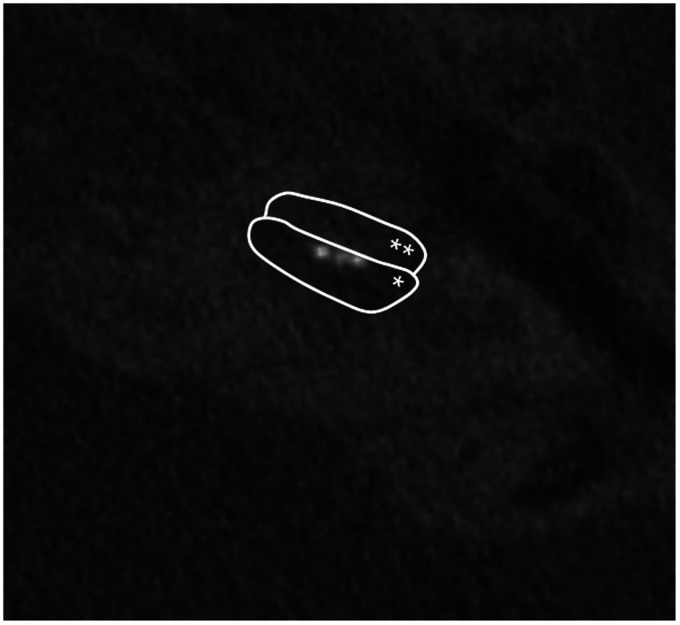
DVT Scan of S119, Indicating a Scalar Dislocation at an Insertion Angle of Approximately 180° to 200°, Corresponding to an Approximate Insertion Depth of 12 to 15 mm. The borders of the first (*) and second (**) cochlear turn are circled for clarity.

**Figure 3.**
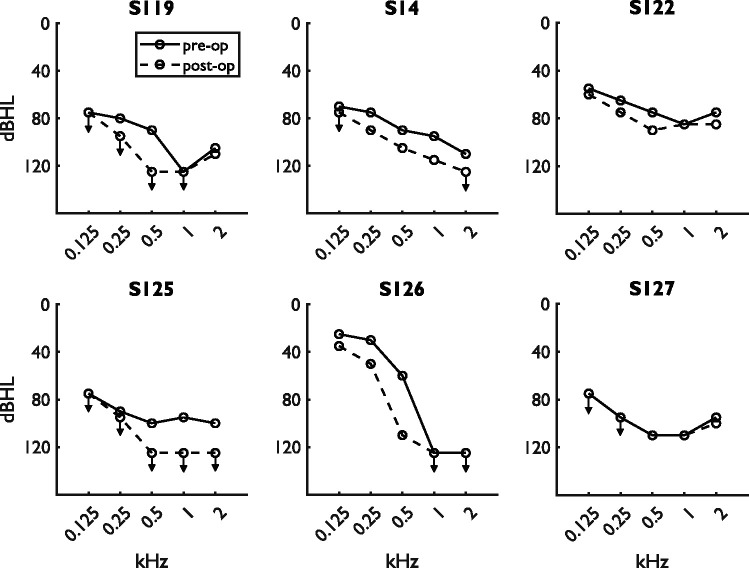
Pre- and Postoperative Audiograms for All Six Subjects. Note that only the solid line is visible if thresholds were unchanged. Thresholds beyond the limits of the audiometer are marked with arrows.

Six subjects were included in this study (S119, S14, S122, S125, S126, and S127). Demographic, audiologic, and radiologic data for all six subjects are summarized in Table 1. Only the DVT scan of S119 showed signs of a possible scalar dislocation (see Figure 2). For all other subjects, a correct positioning of the electrode array within the scala tympani was confirmed based on visual inspection. In S122, four electrodes were located outside of the cochlea on the postoperative CT scan although a full insertion was achieved during surgery. In retrospect, video recordings of the surgery could confirm that the electrodes shifted between the last ECochG recording and the postoperative CT scan. Hence, the insertion angle mentioned in Table 1 does not correspond to the recording location of the final ECochG measurement. The pre- and postoperative hearing thresholds of all subjects are summarized in Table 2 and shown in Figure 3. Subjects S119 and S125 showed complete loss of residual hearing after surgery, and subjects S14 and S126 showed partial preservation. Complete preservation of residual hearing was achieved for subjects S122 and S127.

**Table 1.** Demographic, Audiologic, and Radiologic Data for All Six Subjects.

Subject	Age (years)	Sex	Hearing preservation	CT: sign for scalar dislocation	CT: insertion angle
S119	70	Male	No preservation	Yes	400°
S14	26	Male	Partial preservation	No	410°
S122	76	Male	Complete preservation	No	300°
S125	55	Male	No preservation	No	390°
S126	76	Female	Partial preservation	No	370°
S127	68	Female	Complete preservation	No	450°

*Note.* For S122, the electrode array migrated between the last ECochG recording and the postoperative CT scan; hence, the mentioned insertion angle does not correspond to the insertion depth achieved during surgery. CT = computed tomography.

**Table 2.** Pre- and Postoperative Pure-Tone Average for All Six Subjects, Calculated as the Average of the Threshold Values at 0.125, 0.25, 0.5, 1, and 2 kHz.

Subject	Preoperative PTA, operated side (dB HL)	Postoperative PTA, operated side (dB HL)	Preoperative PTA at 500 Hz	Postoperative PTA at 500 Hz	Preoperative PTA, contralateral side (dB HL)	Postoperative PTA, contralateral side (dB HL)
S119	95	106 (+11)	90	No response (+35)	86	85 (−1)
S14	88	102 (+14)	90	105 (+15)	96	94 (−2)
S122	71	79 (+8)	75	90 (+15)	62	63 (+1)
S125	92	109 (+17)	100	No response (+15)	76	77 (+1)
S126	73	89 (+16)	60	110 (+50)	51	52 (+1)
S127	97	98 (+1)	110	110 (+0)	98	98 (+0)

PTA = pure-tone average.

**Figure 4.**
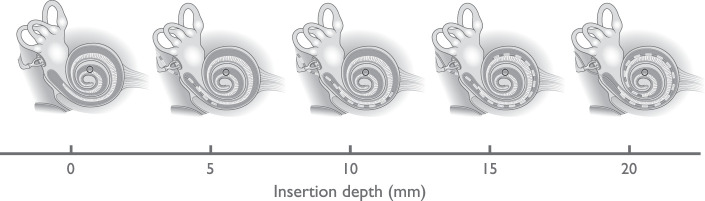
Visualization of the CI’s Positioning for Different Insertion Depths With Respect to the 500 Hz Resonance Location Within the Cochlea (Marked by the Black Circle).

For all subjects, either four or five simultaneous ECochG measurements were obtained during insertion. For four of six subjects (S14, S122, S125, and S127), the intra- and extracochlear sum and difference curves were clearly distinguishable above the noise floor (except for the initial intracochlear sum curve in S14 and the initial intracochlear difference curve in S125). For S119, while clear difference curves were present initially, the intra- and extracochlear sum curves were not clearly distinguishable above the noise floor (except for one intracochlear recording). During the first three quarters of the insertion, S126 showed clear extracochlear recordings, but the intracochlear recordings were within the noise floor. The insertion depths at which an ECochG response was measured varied between subjects, but the first and last simultaneous measurements were always obtained at insertion depths of 2 mm and at full insertion (20 mm), respectively. Figure 4 shows the approximate positioning of the CI for different insertion depths with respect to the location within the cochlea where the basilar membrane’s best frequency is 500 Hz (the acoustic stimulation frequency).

### Difference Curves

**Figure 5.**
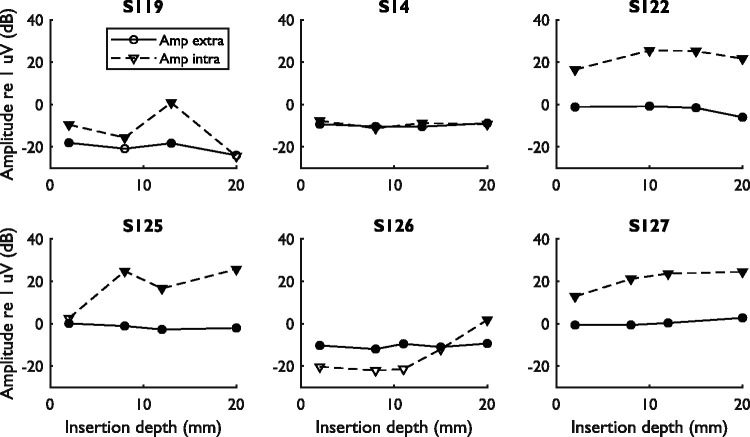
Amplitude Component at 500 Hz of the Extra- and Intracochlear Difference Curves for All Subjects. Data are shown for all simultaneous measurements, starting from the measurement at an insertion depth of 2 mm and ending with the final measurement at full insertion. Measurements within the noise floor are represented by open symbols, while measurements above the noise floor are represented by filled symbols. The amplitude of the extracochlear noise floor was −25.4, −30.3, −35.0, −29.2, −22.8, and −32.3 dB re 1 uV for S119, S14, S122, S125, S126, and S127, respectively. The respective amplitudes for the intracochlear noise floor were −16.2, −26.0, −9.2, 2.5, −6.2, and −5.1 dB re 1 uV.

**Figure 6.**
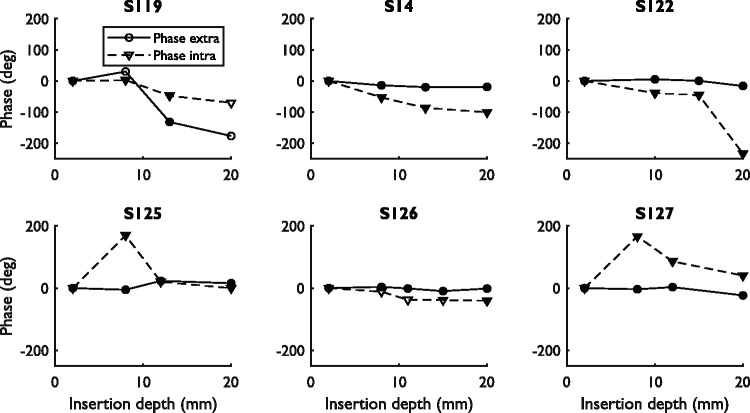
Phase Component at 500 Hz of the Extra- and Intracochlear Difference Curves for All Subjects. The phases determined are not corrected for the cycle. Measurements within the noise floor are represented by open symbols, while measurements above the noise floor are represented by filled symbols.

Figures 5 and 6 show the amplitudes and phases of the difference curves at 500 Hz measured using the extra- and intracochlear electrodes. In the first simultaneous measurement, the amplitudes of the extracochlear difference curve were between 0.12 and 1.0 µV (−18 and 0 dB re 1 µV) while the amplitudes of the intracochlear difference curve were between 0.10 and 6.7 µV (−12 and 16 dB re 1 µV), assessed over all subjects. In all but one of the cases (S126), the amplitude of this initial measurement was larger for the intracochlear signal than for the extracochlear signal. The extracochlear response amplitude showed a relevant decrease between the first and last measurement for S119 (−5.9 dB) and S122 (−4.9 dB). The phases of the extracochlear signals remained stable during insertion for all subjects except S119. The phase change for S119 occurred with amplitude changes that caused the signal to fluctuate around the noise floor, which might explain the phase change. However, it also has to be noted that the phase change occurred at the approximate location of the presumed scalar translocation, suggesting a possible relation between extracochlear phase changes and scalar translocation. The intracochlear signals of all subjects showed large amplitude variations during CI insertion and either slowly progressing phase changes (S119, S14, and S126) or abrupt, near 180° phase changes (S122, S125, and S127).

**Figure 7.**
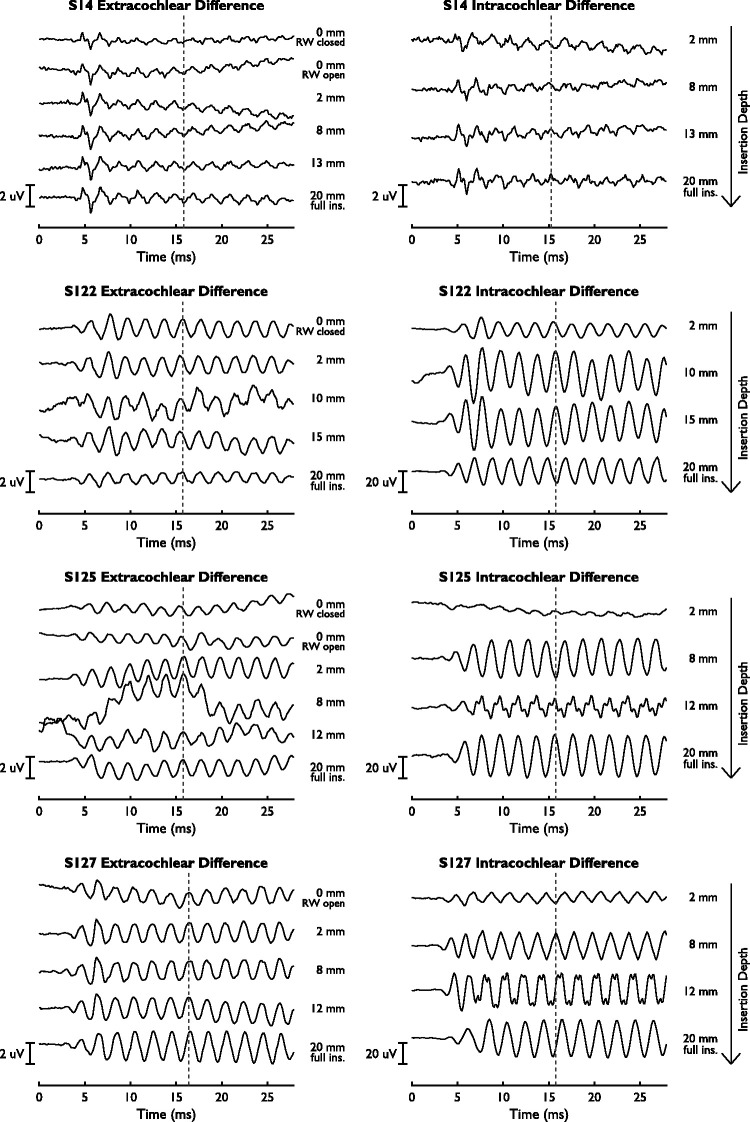
Extra- and Intracochlear Difference Curves for Four Subjects, S14, S122, S125, and S127. Note the differences in scale for the different figures.

The intra- and extracochlear difference curves for one case with a slowly progressing intracochlear phase change (S14) and all cases with abrupt, near 180° phase changes are shown in Figure 7. For S14, there was only a small difference in amplitude between intra- and extracochlear responses, while the amplitudes of the intracochlear responses are much larger than the extracochlear response amplitudes for S122, S125, and S127. For S122, the near 180° phase shift (or phase inversion) in the intracochlear response happened near the end of the insertion and was accompanied by an amplitude drop in the extracochlear response (−3.6 dB relative to the previous measurement). For S125, the phase inversion in the intracochlear response happened during the first half of insertion. Afterward, the recorded signal reversed at an insertion depth of 12 mm accompanied by an amplitude drop and harmonic distortion. At the end of the insertion, the amplitude increased again and the harmonic distortion disappeared. A similar pattern was observed in S127, who showed an early phase inversion that recovered later during insertion, associated with harmonic distortion at an insertion depth of 12 mm.

### Sum Curves

**Figure 8.**
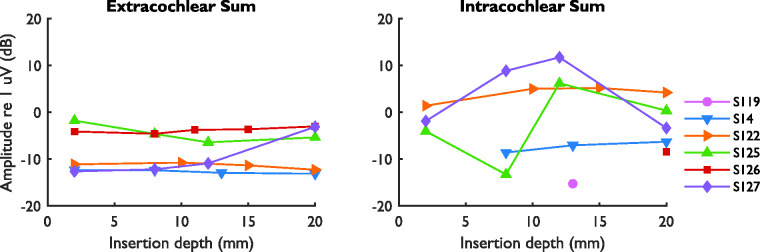
Amplitude Components at 1000 Hz of the Extra- and Intracochlear Sum Curves During Simultaneous Measurements for All Subjects. Amplitudes are shown only for signals that were above the noise floor. The amplitude of the extracochlear noise floor was −46.9, −36.0, −41.3, −35.2, −31.5, and −33.0 dB re 1 uV for S119, S14, S122, S125, S126, and S127, respectively. The respective amplitudes for the intracochlear noise floor were −31.2, −29.0, −28.0, −30.3, −30.3, and −34.5 dB re 1 uV.

**Figure 9.**
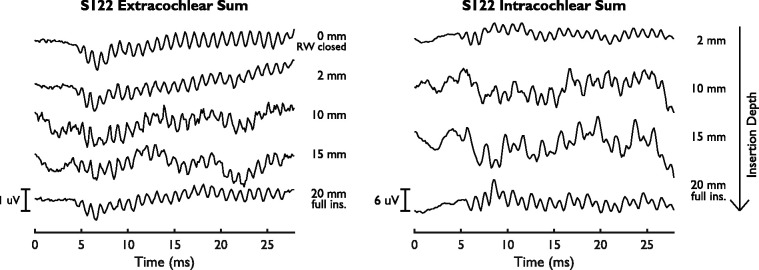
Extra- and Intracochlear Sum Curves for S122.

Figure 8 shows the amplitude tracks of the sum curves. No clear signal could be distinguished in the intra- and extracochlear sum curves of S119 (except for one intracochlear recording) and in the intracochlear sum curves of S126 (except for one recording). The initial amplitudes of the extracochlear sum curves for each subject were between 0.23 and 0.81 µV (−13 and −2 dB re 1 µV), excluding S119, while the amplitudes of the intracochlear sum curves were between 0.06 and 1.18 µV (−24 and 1 dB re 1 µV), excluding S119 and S126. Between the first and last measurement, the extracochlear sum curves showed a relevant decrease for S125 (−3.6 dB) and an increase for S127 (+9.5 dB). For all other subjects, the amplitudes remained stable. As also observed for the difference curves, the intracochlear sum curves showed larger amplitude variations during insertion than the extracochlear sum curves. For S125 and S127, the intracochlear amplitude showed a large increase near an insertion depth of 12 mm and decreased again toward the end of insertion. It should be noted that S125 and S127 were also the cases with early reversible phase shifts of approximately 180°, accompanied by an amplitude drop and harmonic distortion in the difference curves. In some cases (S122, S125, and S126), the intracochlear sum curves showed more noise than the extracochlear sum curves. An exemplary case of intra- and extracochlear sum curves is shown in Figure 9.

## Discussion

The aim of this study was to compare simultaneously recorded intra- and extracochlear ECochG signals to gain a better understanding of the mechanisms causing intracochlear signal changes. Specifically, it was intended to identify the causes behind intracochlear ECochG response characteristics, such as phase changes and harmonic distortions in the difference curves, and whether the presence of these characteristics can help distinguish response amplitude decreases caused by changes in cochlear functionality and amplitude decreases resulting solely from a change in recording location. Hereby, this study could help to enable the use of intracochlear ECochG alone for intra-operative monitoring.

### Intra- Versus Extracochlear ECochG

In the initial simultaneous measurement, the amplitude of the intracochlear difference curve was larger than the amplitude of the corresponding extracochlear curve for all subjects except S126. For the sum curves, this was true in three of the six cases. In a recent study (Dalbert, Sijgers, et al., 2020) in which we investigated simultaneous recordings in atraumatic insertions with a different recording setup for intracochlear recordings, we found intracochlear difference curves to be larger in all cases. Therefore, we assume that intracochlear difference curves should be larger in all simultaneous recordings and that there may have been a problem with the connection between the CI’s most apical electrode and the reference ring electrode in S126, causing the smaller intracochlear responses. The most likely underlying reason that difference curves recorded from an intracochlear location are usually larger than simultaneously recorded extracochlear signals is that the recording electrode is closer to the hair cell signal generators and the difference curve is thought to be mainly generated by the hair cells. This proximity is less straightforward for the sum recordings, which largely result from neural potentials within the modiolus and could thus be larger in either intra- or extracochlear recordings.

In most recordings, the intracochlear difference curves were less susceptible to noise than the extracochlear difference curves, whereas the intracochlear sum curves were affected by noise that was not observed in extracochlear recordings in three of the six subjects. Harris, Riggs, Koka, et al. (2017) and Giardina et al. (2019) have published the only studies so far that used a similar clip setup to measure intracochlear ECochG signals. Giardina et al. also observed increased line noise in ECochG recordings acquired through the clip electrode and suggested that the high-impedance pathway between the external amplifier and the apical electrode array contact was a possible cause. This seems to regularly be the case in an intracochlear recording setup such as the one presented here and therefore represents a limitation of the described method. The noise represents more of a problem in the sum curves than in the difference curves because the signals in the difference curves are usually much larger than in the sum curves and therefore more noise resilient.

### Intracochlear Phase Changes

Phase inversions in the intracochlear difference curves occurred in three cases (S122, S125, and S127). For the other three subjects, slowly progressing phase changes were observed. Slowly progressing phase changes can be explained by the phase lag of the hair cell vibrations in the vicinity of the recording electrode for more apical recording locations (Campbell et al., 2017; Gundersen et al., 1978; v. Békésy, 1953), as the CM’s phase reflects the phase of the basilar membrane’s traveling wave (Tasaki et al., 1952). However, phase inversions in ECochG responses cannot be explained by this. For phase inversions, three explanations have been proposed in the literature. First, CM recordings from the scala vestibuli have a polarity opposite that for scala tympani recordings (Davis et al., 1950) caused by the hair cells acting as an electrical dipole (Hudspeth, 1982). Phase inversions could therefore indicate scalar dislocations. Second, movement of the recording electrode past the location of resonance on the basilar membrane could lead to phase inversions (Kohllöffel, 1970). Third, interference of hair cell responses from different parts of the cochlea or interference between hair cell and neural components could cause sudden changes in phase (Giardina et al., 2019), although these phase changes would not necessarily be 180°.

In this study, the subjects with phase inversions had extra- and intracochlear difference curves with higher amplitudes than the subjects with slowly progressing phase changes. In addition, the amplitude differences between intra- and extracochlear difference signals were larger for the measurements demonstrating phase inversions. This could indicate that these subjects had more intact hair cell populations compared with subjects showing slowly progressing phase changes, and different hair cell populations could have dominated the recordings at different insertion depths. The data from this study do not show a clear relationship between the pre- or postoperative audiogram and the observed phase changes. It seems unlikely that a scalar dislocation could have caused phase inversions in any of the measurements, as the CT scans of subjects with phase inversions indicated a correct scala tympani insertion, and residual hearing was fully preserved in S122 and S127. In addition, the phase change is transient in S125 and S127. This is in agreement with a study by Koka et al. (2018) in which 180° phase shifts were measured only for scala tympani insertions.

It is interesting to have a closer look at the results of the one subject (S119) in whom a scalar dislocation was suspected based on postinsertion imaging results. Although the intracochlear difference curves showed only slowly progressing phase changes during insertion, which is in line with our expectations based on the study by Koka et al. (2018), the extracochlear difference curves did show a 180° phase shift near the location of suspected translocation. This phase shift cannot be explained by any of the previously suggested mechanisms behind intracochlear phase inversions and to our knowledge, there is no known extracochlear ECochG signature for translocation. The results of this study therefore suggest that the phase of the extracochlear signal may be a relevant topic for further research.

When investigating the intracochlear phase inversions with respect to their recording locations, two patterns were observed: (a) early phase inversions between 2 and 8 mm insertion depth associated with harmonic distortion at an insertion depth of 12 mm (S125 and S127) and (b) phase inversions near the end of insertion (S122, S125, and S127). The early phase inversions in S125 and S127 were accompanied by large amplitude increases in the sum curves and harmonic distortion in the difference curves. Such amplitude increases in the sum curves were not observed in subjects without early phase inversions. The amplitudes decreased again toward the end of insertion in both S125 and S127. This indicates that distortions between CM and ANN signals may have caused the early phase shift, as suggested by Giardina et al. (2019). Phase inversions near the end of insertion were recorded in the vicinity of the 500 Hz resonance point. Therefore, signals from the 500 Hz resonance point may have suddenly dominated the recording from the intracochlear electrode at this final recording point and caused the phase inversion. In S125 and S127, the phase inversion toward the end of the insertion could of course also be due to a reduction of the large ANN contribution earlier, a reversal of the mechanism discussed for the early phase inversions.

However, the data of S125 and S127 suggest that the phase inversions could also have occurred because the recording electrode moves around an electric dipole. Two observations in the difference curves of these subjects are characteristic of dipole behavior: (a) the phase inversion between 2 and 8 mm insertion depth is accompanied by a large amplitude increase and (b) the phase inversion between 8 and 20 mm seems to be accompanied by an amplitude decrease in the CM signals at 12 mm insertion depth, which is especially strong in S125. This amplitude decrease may have caused the ANN to dominate the recordings, leading to harmonic distortions. Although phase inversions due to dipole behavior have previously been suggested to reflect a change in measurement location to a different scala (Davis et al., 1950), which does not seem to be the case in our data, the complex electrical properties of the cochlea make the spread of the electric field resulting from the hair cell dipole difficult to predict (Hudspeth, 1982). Our data therefore suggest that dipole behavior may occur even with same-scalar insertions, although further research will be needed to confirm this.

### Interpretation of Intracochlear Amplitude Drops

For the eventual use of intracochlear ECochG measurements as an insertion monitoring tool, an understanding of the relationship between amplitude drops and intracochlear damage or hearing outcomes is essential. The results of this study indicate that an early amplitude drop in the intracochlear difference response is not accompanied by a drop in the extracochlear difference amplitude. The intracochlear amplitude drop is likely caused by interference between CM and ANN components, or by a decrease in CM amplitude due to dipole behavior, causing the ANN to be represented more strongly in the difference curves. Therefore, early intracochlear difference curve amplitude decreases accompanied by large, near 180° phase shifts, harmonic distortion, or large increases in sum signal amplitude are likely not indicative of trauma. Later amplitude decreases in intracochlear difference responses (near full insertion) did correspond to extracochlear amplitude drops in this study (S119 and S122), regardless of whether these drops were accompanied by phase inversions (S122) or not (S119). These amplitude drops may therefore correspond to cochlear trauma, although no relationship with preservation of residual hearing was found in this small cohort. It is reassuring that the studies of Giardina et al. (2019) and Koka et al. (2018), who compared intracochlear ECochG signals with hearing outcomes and CI positioning, reached similar conclusions regarding the interpretation of early and late amplitude drops.

## Conclusion

Decreased amplitudes in intracochlear ECochG recordings in the early phase of the insertion associated with phase shifts and harmonic distortion can be observed without associated amplitude changes in extracochlear recordings. Such decreases in amplitude are likely caused by movement of the recording electrode with respect to the different signal generators. Amplitude drops toward the end of the insertion, with or without phase shift and without harmonic distortion, are reflected in extracochlear ECochG recordings. Comparison of intracochlear ECochG recordings with simultaneous extracochlear recordings could help to differentiate between atraumatic and traumatic changes in intracochlear ECochG responses.

## Data Accessibility Statement

The data that support the findings of this study are available from the corresponding author upon reasonable request.

## References

[bibr1-2331216521990594] Acharya A. N. Tavora-Vieira D. Rajan G. P. (2016). Using the implant electrode array to conduct real-time intraoperative hearing monitoring during pediatric cochlear implantation: Preliminary experiences. Otology & Neurotology, 37(2), e148–e153. 10.1097/Mao.0000000000000950 26756149

[bibr2-2331216521990594] Adunka O. F. Giardina C. K. Formeister E. J. Choudhury B. Buchman C. A. Fitzpatrick D. C. (2016). Round window electrocochleography before and after cochlear implant electrode insertion. Laryngoscope, 126(5), 1193–1200. 10.1002/lary.25602 26360623 PMC5949050

[bibr3-2331216521990594] Balkany T. J. Connell S. S. Hodges A. V. Payne S. L. Telischi F. F. Eshraghi A. A., Angeli, S. I., Germani, R., Messiah, S., & Arheart K. L. (2006). Conservation of residual acoustic hearing after cochlear implantation. Otology & Neurotology, 27(8), 1083–1088. 10.1097/01.mao.0000244355.34577.85 17130798

[bibr4-2331216521990594] Bester C. W. Campbell L. Dragovic A. Collins A. O’Leary S. J. (2017). Characterizing electrocochleography in cochlear implant recipients with residual low-frequency hearing. Frontiers in Neuroscience, 11, 141. 10.3389/fnins.2017.00141 28386212 PMC5363175

[bibr5-2331216521990594] Calloway N. H. Fitzpatrick D. C. Campbell A. P. Iseli C. Pulver S. Buchman C. A. Adunka O. F. (2014). Intracochlear during cochlear implantation. Otology & Neurotology, 35(8), 1451–1457. 10.1097/mao.0000000000000451 24892369

[bibr6-2331216521990594] Campbell L. Bester C. Iseli C. Sly D. Dragovic A. Gummer A. W. O’Leary S. (2017). Electrophysiological evidence of the basilar-membrane travelling wave and frequency place coding of sound in cochlear implant recipients. Audiology and Neurotology, 22(3), 180–189. 10.1159/000478692 29084395

[bibr7-2331216521990594] Campbell L. Kaicer A. Briggs R. O’Leary S. (2015). Cochlear response telemetry: Intracochlear electrocochleography via cochlear implant neural response telemetry pilot study results. Otology & Neurotology, 36(3), 399–405. 10.1097/mao.0000000000000678 25473960

[bibr8-2331216521990594] Campbell L. Kaicer A. Sly D. Iseli C. Wei B. Briggs R. O’Leary S. (2016). Intraoperative real-time cochlear response telemetry predicts hearing preservation in cochlear implantation. Otology & Neurotology, 37(4), 332–338. 10.1097/mao.0000000000000972 26859542

[bibr9-2331216521990594] Choudhury B. Adunka O. F. Demason C. E. Ahmad F. I. Buchman C. A. Fitzpatrick D. C. (2011). Detection of intracochlear damage with cochlear implantation in a gerbil model of hearing loss. Otology & Neurotology, 32(8), 1370. 10.1097/MAO.0b013e31822f09f2 21921858 PMC3338854

[bibr10-2331216521990594] Dalbert A. Huber A. Veraguth D. Roosli C. Pfiffner F. (2016). Assessment of cochlear trauma during cochlear implantation using electrocochleography and cone beam computed tomography. Otology & Neurotology, 37(5), 446–453. 10.1097/mao.0000000000000998 26945317

[bibr11-2331216521990594] Dalbert A. Pfiffner F. Hoesli M. Koka K. Veraguth D. Roosli C. Huber A. (2018). Assessment of cochlear function during cochlear implantation by extra-and intracochlear electrocochleography. Frontiers in Neuroscience, 12, 18. 10.3389/fnins.2018.00018 29434534 PMC5790789

[bibr12-2331216521990594] Dalbert A. Pfiffner F. Röösli C. Thoele K. Sim J. H. Gerig R. Huber A. M. (2015). Extra- and Intracochlear electrocochleography in cochlear implant recipients. Audiology and Neurotology, 20(5), 339–348. 10.1159/000438742 26340649

[bibr13-2331216521990594] Dalbert A. Rohner P. Roosli C. Veraguth D. Huber A. Pfiffner F. (2020). Correlation between electrocochleographic changes during surgery and hearing outcome in cochlear implant recipients: A case report and systematic review of the literature. Otology & Neurotology, 41(3), 318–326. 10.1097/mao.0000000000002506 31834213

[bibr14-2331216521990594] Dalbert A. Sijgers L. Grosse J. Veraguth D. Roosli C. Huber A. Pfiffner F. (2020). Simultaneous intra-and extracochlear electrocochleography during electrode insertion. Ear and Hearing. Advance online publication. 10.1097/AUD.0000000000000935 32826509

[bibr15-2331216521990594] Dalbert A. Sim J. H. Gerig R. Pfiffner F. Roosli C. Huber A. (2015). Correlation of electrophysiological properties and hearing preservation in cochlear implant patients. Otology & Neurotology, 36(7), 1172–1180. 10.1097/mao.0000000000000768 25839980

[bibr16-2331216521990594] Davis H. Fernandez C. Mc A. D. (1950). The excitatory process in the cochlea. Proceedings of the National Academy of Sciences of the United States of America, 36(10), 580–587. 10.1073/pnas.36.10.580 14808143 PMC1063246

[bibr17-2331216521990594] DeMason C. Choudhury B. Ahmad F. Fitzpatrick D. C. Wang J. Buchman C. A. Adunka O. F. (2012). Electrophysiological properties of cochlear implantation in the gerbil using a flexible array. Ear and Hearing, 33(4), 534–542. 10.1097/AUD.0b013e3182498c28 22436408 PMC3613224

[bibr18-2331216521990594] Forgues M. Koehn H. A. Dunnon A. K. Pulver S. H. Buchman C. A. Adunka O. F. Fitzpatrick D. C. (2014). Distinguishing hair cell from neural potentials recorded at the round window. Journal of Neurophysiology, 111(3), 580–593. 10.1152/jn.00446.2013 24133227 PMC3921406

[bibr19-2331216521990594] Giardina C. K. Brown K. D. Adunka O. F. Buchman C. A. Hutson K. A. Pillsbury H. C. Fitzpatrick D. C. (2019). Intracochlear electrocochleography: Response patterns during cochlear implantation and hearing preservation. Ear and Hearing, 40(4), 833–848. 10.1097/aud.0000000000000659 30335669 PMC6534483

[bibr20-2331216521990594] Giardina C. K. Khan T. E. Pulver S. H. Adunka O. F. Buchman C. A. Brown K. D., . . . Fitzpatrick D. C. (2018). Response changes during insertion of a cochlear implant using extracochlear electrocochleography. Ear and Hearing, 39(6), 1146–1156. 10.1097/aud.0000000000000571 29554036 PMC6139286

[bibr21-2331216521990594] Gundersen T. Skarstein O. Sikkeland T. (1978). A study of the vibration of the basilar membrane in human temporal bone preparations by the use of the Mössbauer effect. Acta Otolaryngologica, 86(3-4), 225–232. 10.3109/00016487809124740 707065

[bibr22-2331216521990594] Harris M. S. Riggs W. J. Giardina C. K. O’Connell B. P. Holder J. T. Dwyer R. T., . . . Adunka O. F. (2017). Patterns seen during electrode insertion using intracochlear electrocochleography obtained directly through a cochlear implant. Otology & Neurotology, 38(10), 1415–1420. 10.1097/mao.0000000000001559 28953607 PMC5920552

[bibr23-2331216521990594] Harris M. S. Riggs W. J. Koka K. Litvak L. M. Malhotra P. Moberly A. C., . . . Adunka O. F. (2017). Real-time intracochlear electrocochleography obtained directly through a cochlear implant. Otology & Neurotology, 38(6), e107–e113. 10.1097/mao.0000000000001425 28498269

[bibr24-2331216521990594] Haumann S. Imsiecke M. Bauernfeind G. Büchner A. Helmstaedter V. Lenarz T. Salcher R. B. (2019). Monitoring of the inner ear function during and after cochlear implant insertion using electrocochleography. Trends in Hearing, 23, 1–18. 10.1177/2331216519833567 PMC6435875 30909815

[bibr25-2331216521990594] Hudspeth A. J. (1982). Extracellular current flow and the site of transduction by vertebrate hair cells. Journal of Neuroscience, 2(1), 1–10. 10.1523/jneurosci.02-01-00001.1982 6275046 PMC6564293

[bibr26-2331216521990594] Kohllöffel L. (1970). Longitudinal amplitude and phase distribution of the cochlear microphonic (guinea pig) and spatial filtering. Journal of Sound and Vibration, 11(3), 325–334. 10.1016/s0022-460x(70)80036-0

[bibr27-2331216521990594] Koka K. Riggs W. J. Dwyer R. Holder J. T. Noble J. H. Dawant B. M., . . . Labadie R. F. (2018). Intra-cochlear electrocochleography during cochear implant electrode insertion is predictive of final scalar location. Otology & Neurotology, 39(8), e654–e659. 10.1097/mao.0000000000001906 30113557 PMC6097527

[bibr28-2331216521990594] Mandalà M. Colletti L. Tonoli G. Colletti V. (2012). Electrocochleography during cochlear implantation for hearing preservation. Otolaryngology—Head and Neck Surgery, 146(5), 774–781. 10.1177/0194599811435895 22291043

[bibr29-2331216521990594] O’Connell B. P. Holder J. T. Dwyer R. T. Gifford R. H. Noble J. H. Bennett M. L., . . . Labadie R. F. (2017). Intra-and postoperative electrocochleography may be predictive of final electrode position and postoperative hearing preservation. Frontiers in Neuroscience, 11, 291. 10.3389/fnins.2017.00291 28611574 PMC5447029

[bibr30-2331216521990594] Radeloff A. Shehata-Dieler W. Scherzed A. Rak K. Harnisch W. Hagen R. Mlynski R. (2012). Intraoperative monitoring using cochlear microphonics in cochlear implant patients with residual hearing. Otology & Neurotology, 33(3), 348–354. 10.1097/MAO.0b013e318248ea86 22377649

[bibr31-2331216521990594] Ramos-Macias A. O’Leary S. Ramos-deMiguel A. Bester C. Falcon-González J. C. (2019). Intraoperative intracochlear electrocochleography and residual hearing preservation outcomes when using two types of slim electrode arrays in cochlear implantation. Otology & Neurotology, 40(5S Suppl 1), s29–s37. 10.1097/mao.0000000000002212 31225820

[bibr32-2331216521990594] Riggs W. J. Dwyer R. T. Holder J. T. Mattingly J. K. Ortmann A. Noble J. H., . . . Adunka O. F. (2019). Intracochlear electrocochleography: Influence of scalar position of the cochlear implant electrode on postinsertion results. Otology & Neurotology, 40(5), e503–e510. 10.1097/mao.0000000000002202 31083085 PMC6530483

[bibr33-2331216521990594] Scott W. C. Giardina C. K. Pappa A. K. Fontenot T. E. Anderson M. L. Dillon M. T., . . . Fitzpatrick D. C. (2016). The compound action potential in subjects receiving a cochlear implant. Otology & Neurotology, 37(10), 1654–1661. 10.1097/mao.0000000000001224 27749750 PMC5242224

[bibr34-2331216521990594] Tasaki I. Davis H. Legouix J. P. (1952). The space‐time pattern of the cochlear microphonics (guinea pig), as recorded by differential electrodes. Journal of the Acoustical Society of America, 24(5), 502–519. 10.1121/1.1906928

[bibr35-2331216521990594] v. Békésy G. (1953). Description of some mechanical properties of the organ of Corti. Journal of the Acoustical Society of America, 25(4), 770–785. 10.1121/1.1907174

